# Extract from *Dioscorea bulbifera* L. rhizomes aggravate pirarubicin-induced cardiotoxicity by inhibiting the expression of P-glycoprotein and multidrug resistance-associated protein 2 in the mouse liver

**DOI:** 10.1038/s41598-021-99264-2

**Published:** 2021-10-05

**Authors:** Li-rui Sun, Qiu-shi Guo, Wei Zhou, Min Li

**Affiliations:** 1 Department of Pharmacy, The First Hospital of Jilin University, Changchun, Jilin China; 2 Pharmacological Experiment Center, School of Pharmaceutical Sciences, Jilin University, Changchun, Jilin China

**Keywords:** Pharmacodynamics, Pharmacokinetics

## Abstract

Chinese herbal medicine is widely used because it has a good safety profile and few side effects. However, the risk of adverse drug reactions caused by herb-drug interactions (HDIs) is often overlooked. Therefore, the task of identifying possible HDIs and elucidating their mechanisms is of great significance for the prevention and treatment of HDI-related adverse reactions. Since extract from *Dioscorea bulbifera* L. rhizomes (DB) can cause various degrees of liver damage, it is speculated that HDIs may occur between DB extract and chemicals metabolized or excreted by the liver. Our study revealed that the cardiotoxicity of pirarubicin (THP) was increased by co-administration of DB, and the expression of P-glycoprotein (P-gp) and multidrug resistance-associated protein 2 (Mrp2) in the liver was inhibited by DB extract, which led to the accumulation of THP in heart tissue. In conclusion, there are risks of the co-administration of DB extract and THP. The mechanism of HDIs can be better revealed by targeting the efflux transporters.

## Introduction

With the popularization of Chinese herbal medicines, the interaction between Chinese herbal medicines and other drugs has become an increasingly important safety concern in the clinical application of traditional medicines^1^. Research in this area is expanding rapidly. The interactions between Chinese herbal medicines and drugs are complex, involving many chemical components and many types of pharmacological activity. The currently known herb-drug interactions (HDIs) occur mainly at the pharmacokinetic and pharmacodynamic levels; in particular, a great deal of research has focused on the pharmacokinetic interactions, in particular, on the absorption, distribution, metabolism and excretion^2,3^. However, studies on the pharmacodynamic interactions between herbs and drugs are still very limited. Herbal medicines can affect drug metabolism by altering the activity of liver enzymes, such as cytochrome P450 (CYP450), and membrane transporters, such as P-glycoprotein (P-gp) and multidrug resistance-associated protein 2 (Mrp2), which may change the pharmacodynamic process of drugs and thus cause drug concentrations to change in vivo^4,5^.

**Figure 1:**
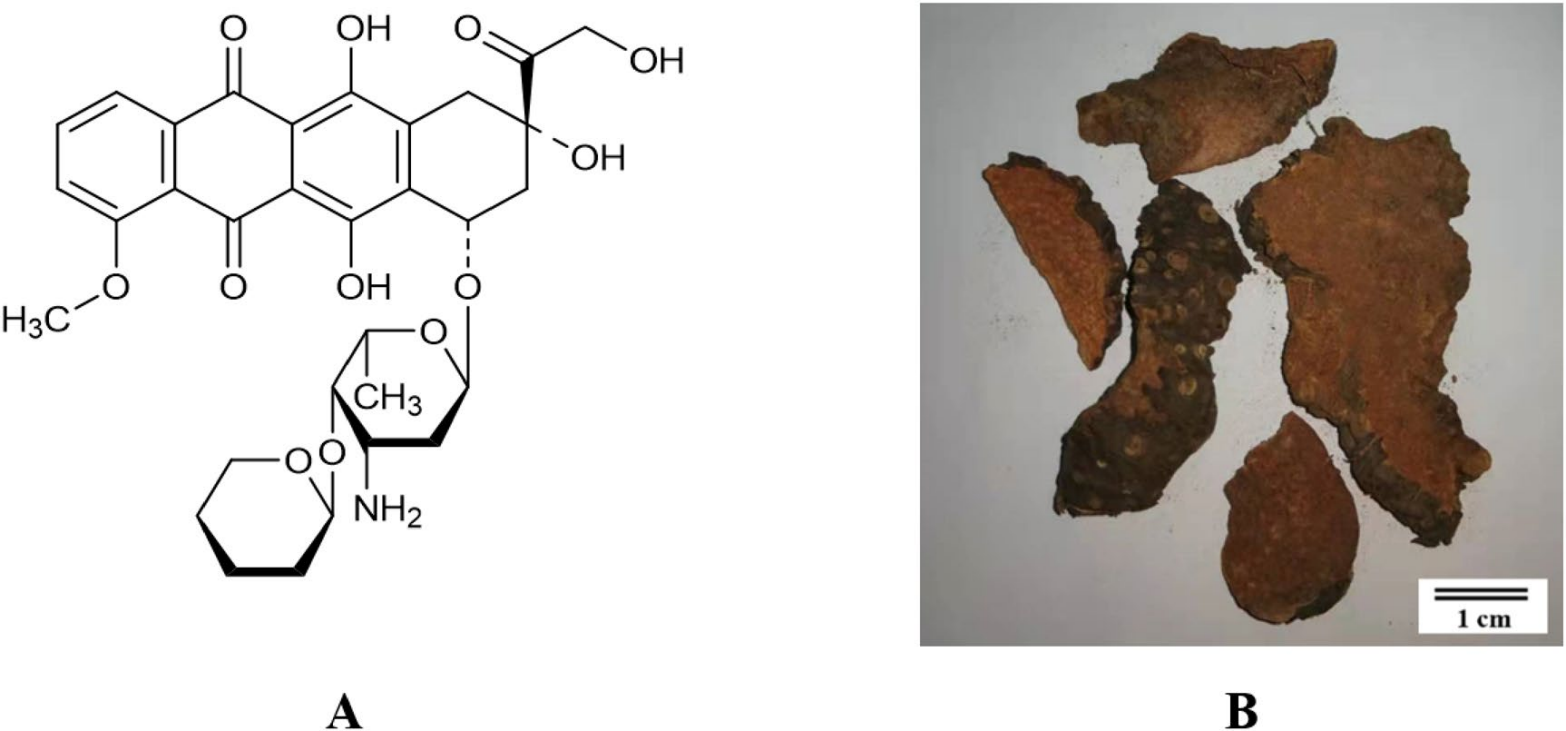
(**A**) Structure of pirarubicin (THP) and (**B**) slices of *Dioscorea bulbifera* L. rhizomes (DB).

Pirarubicin (THP) is an anthracycline that interferes with the synthesis of DNA in tumor cells and is widely used in the treatment of several kinds of tumors (Fig. 1A)^6^. However, the clinical use of THP has been severely limited due to its severe cardiotoxicity, which is characterized by irreversibility and drug accumulation^7^. Liver metabolism and excretion, especially via the efflux transporter P-gp, Mrp2, play an important role in the metabolism of anthracycline; therefore, special attention should be paid to the occurrence of cardiotoxicity when THP is taken alongside drugs that can inhibit cardiac metabolism and drug excretion^8,9^.

*Dioscorea bulbifera* L. is a Chinese herbal medicine that is commonly used in the clinic (Fig. 1B). This herb has a wide range of pharmacological activity. In recent years, the antitumor effect of extract from *Dioscorea bulbifera* L. rhizomes (DB) has received increasing attention; for example, it can inhibit cervical cancer and hepatocarcinoma^10^. DB extract contains a variety of components, including diterpene lactones, steroidal saponins, flavonoids, alkaloids, and micronutrients, among others^11^. DB extract can lead to liver injury and downregulation of the intrahepatic expression of P-gp. These effects may be an important contributor to abnormal biotransformation in the process of drug metabolism^12^. Thus, we hypothesize that DB may increase the cardiotoxicity risk of THP when the two are co-administered. In our study, we explored liver and heart injury in mice and assessed the accumulation of THP, the localization and expression of P-gp and Mrp2 to investigate the mechanisms of the HDI between DB and THP.

## Results

### Effect of DB extract on the cardiotoxicity induced by THP

**Figure 2:**
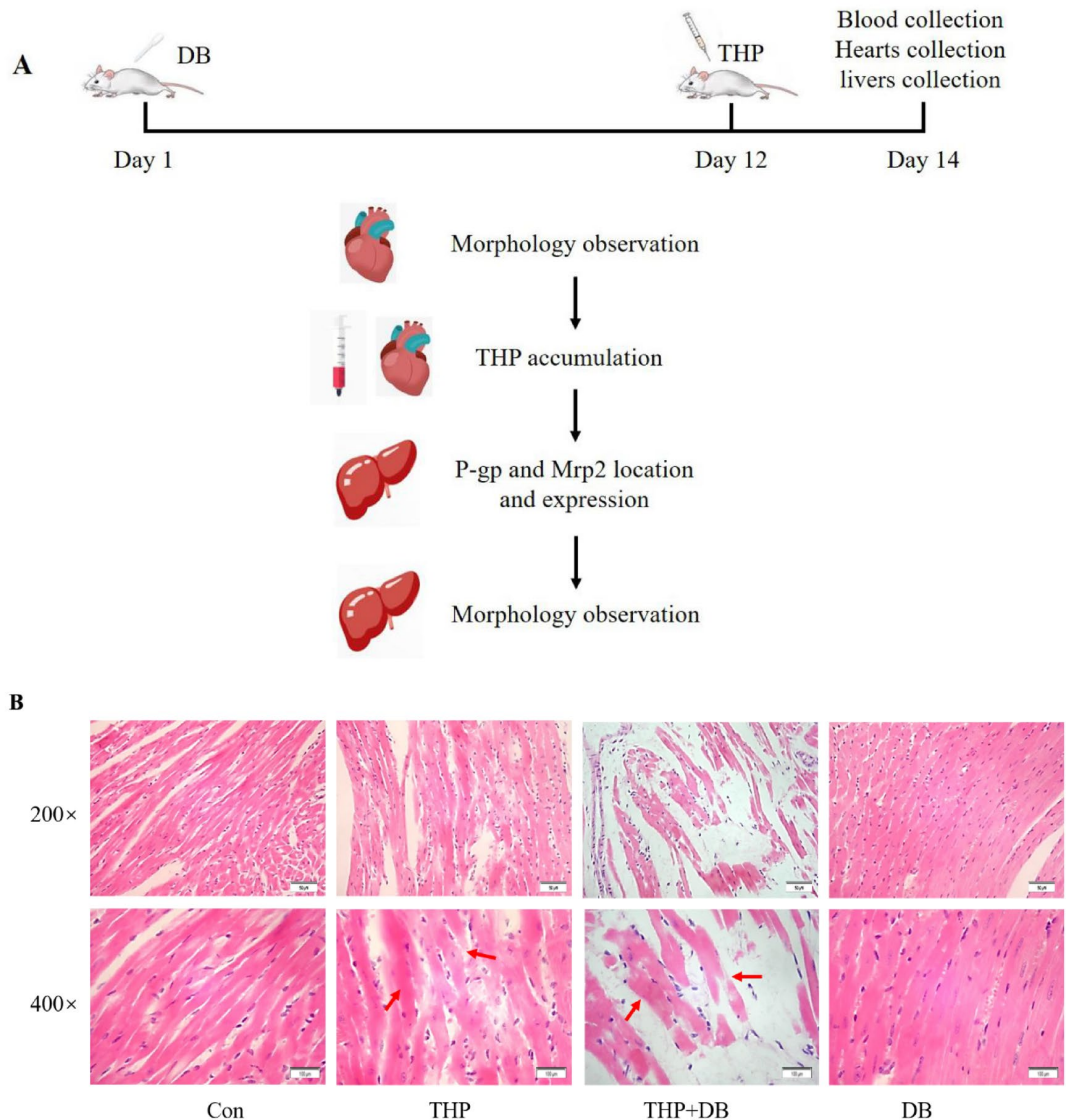
(**A**) The experimental process and procedures. (**B**) Morphology observation images (×200 and ×400) in mice hearts. The hearts of mice were stained with HE. Homogeneous red stain of cardiac myocytes, loose arrangement of cardiac fibers, muscle fiber breakage and obviously light staining were observed in THP + DB group. Cardiac muscle light staining was observed in THP group (the morphological changes were indicated by an arrow). Normal myocardial cell morphology was observed in both of the control group and DB group. Con: Control group, THP: pirarubicin group, THP + DB: pirarubicin and DB extract group, DB: DB extract group.

Heart biopsies from mice treated with THP and DB extract showed homogeneous red stain of cardiac myocytes, loose arrangement of cardiac fibers, muscle fiber breakage and obviously light staining (Fig. 2B). Only cardiac muscle light staining was observed in the THP group, and normal myocardial cell morphology was observed in both of the control group and DB group.

### Effect of DB extract on the accumulation of THP

The precision and accuracy were evaluated at three concentration levels (0.8, 5.0 and 15.0 μg/mL) in 3 replicates. Intraday precisions were analyzed 6 times in one day and interday precisions were analyzed for 6 consecutive days. Intra-day and inter-day precisions used the relative standard deviation (RSD) as indicator (Supplementary Table 1).

**Figure 3:**
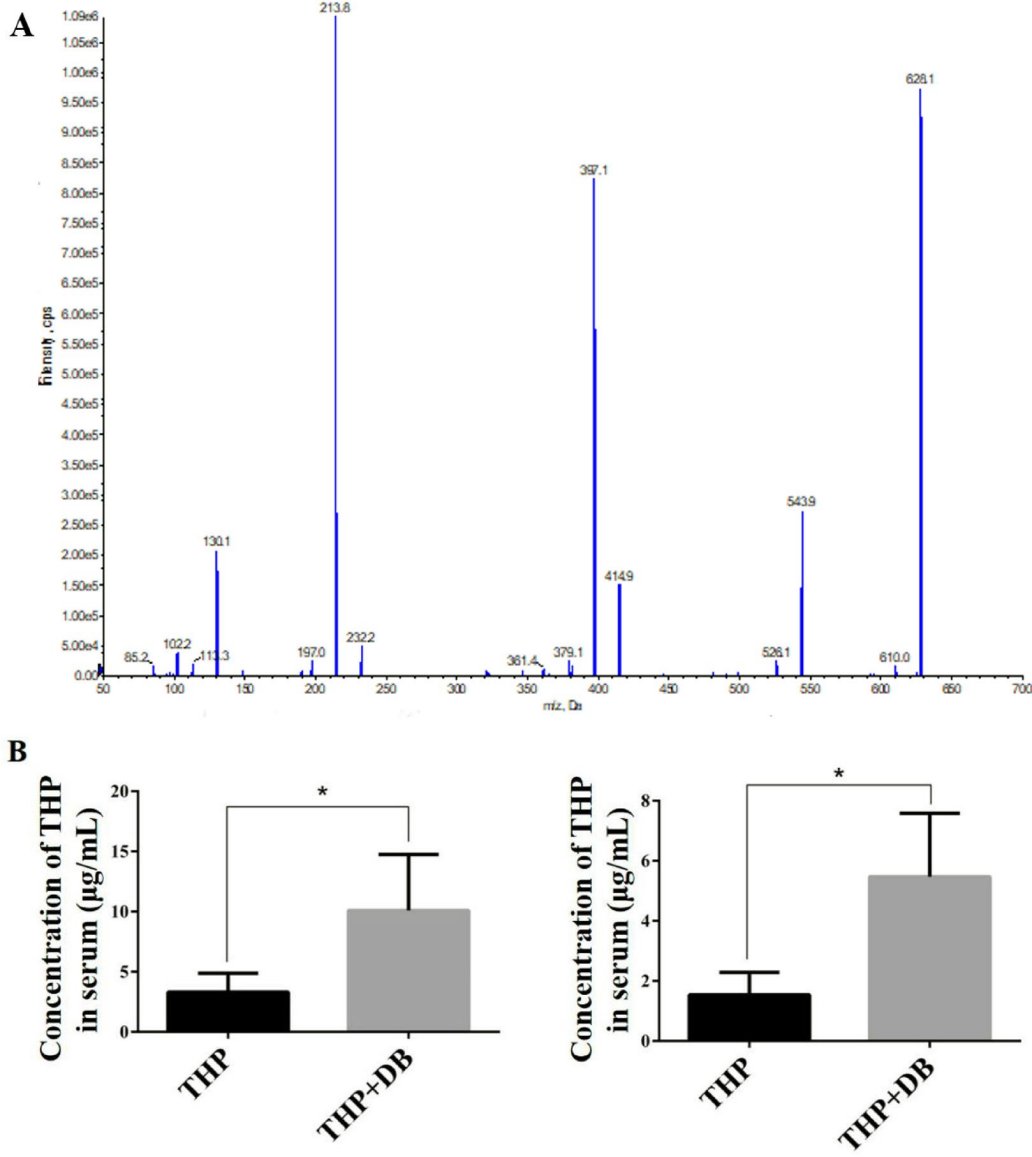
(**A**) The mass spectrogram of THP. (**B**) The concentrations of THP in serum and heart. Blood was collected from mouse eye sockets and the concentrations of THP in serum and heart were detected by HPLC–MS analysis. Differences between groups were assessed by Student’s t-test. n = 8, *p < 0.05.

We detected the THP concentrations in THP and THP + DB groups. In THP + DB group, the concentrations of THP in both tissues were significantly elevated (*p* < 0.05) (Fig. 3).

### Effect of DB extract on the intrahepatic expression of P-gp and Mrp2

**Figure 4:**
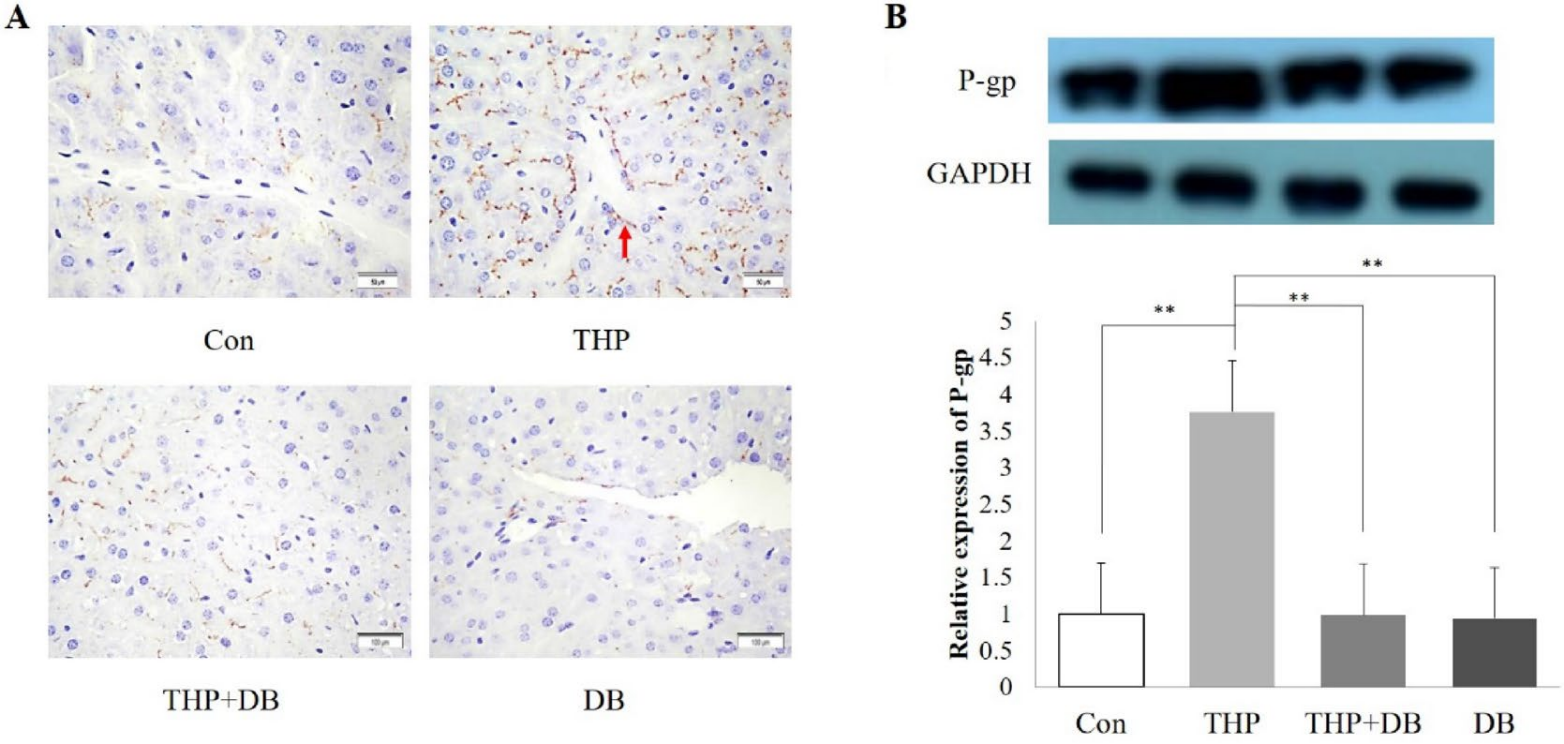
(**A**) Immunohistochemistry section images (×400) of intrahepatic P-gp (the localization of P-gp was indicated by an arrow). The localization of P-gp in liver was detected by immunohistochemistry. (**B**) The P-gp expression in different groups. The P-gp expression of mouse livers was analyzed by Western blot. Statistics were performed with Two-way ANOVA. n = 3, F = 308.599, **p < 0.01, compared with THP group.

**Figure 5:**
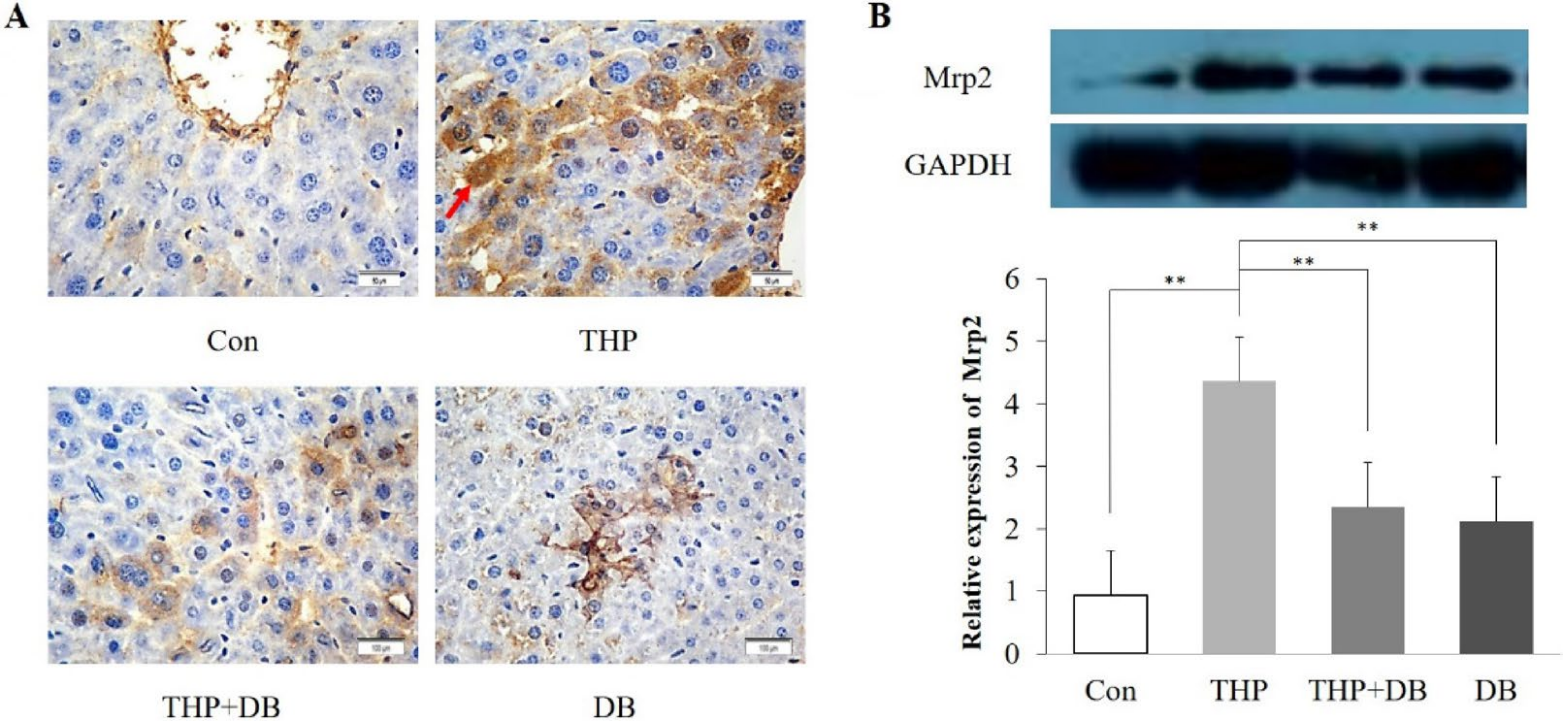
(**A**) Immunohistochemistry section images (×400) of intrahepatic Mrp2 (the localization of Mrp2 was indicated by an arrow). The localization of Mrp2 in liver was detected by immunohistochemistry. (**B**) The Mrp2 expression in different groups. The Mrp2 expression of mouse livers was analyzed by Western blot. Statistics were performed with Two-way ANOVA. n = 3, F = 35.134, **p < 0.01, compared with THP group.

P-gp and Mrp2 membrane localization was found in hepatocytes after THP treatment, which was eliminated by coadministration of THP and DB. As expected, there was no P-gp and Mrp2 membrane localization observed in mice of the DB and control groups (Figs. 4A, 5A).

Similarly, compared to the control group, the levels of P-gp and Mrp2 expressions in mouse livers of THP group were increased by 3.76- and 4.36-fold, respectively (*p* < 0.01). However, there was absent when THP and DB were co-administered (*p* < 0.01) (Figs. 4B, 5B).

### Effect of DB extract on the hepatocytes

**Figure 6:**
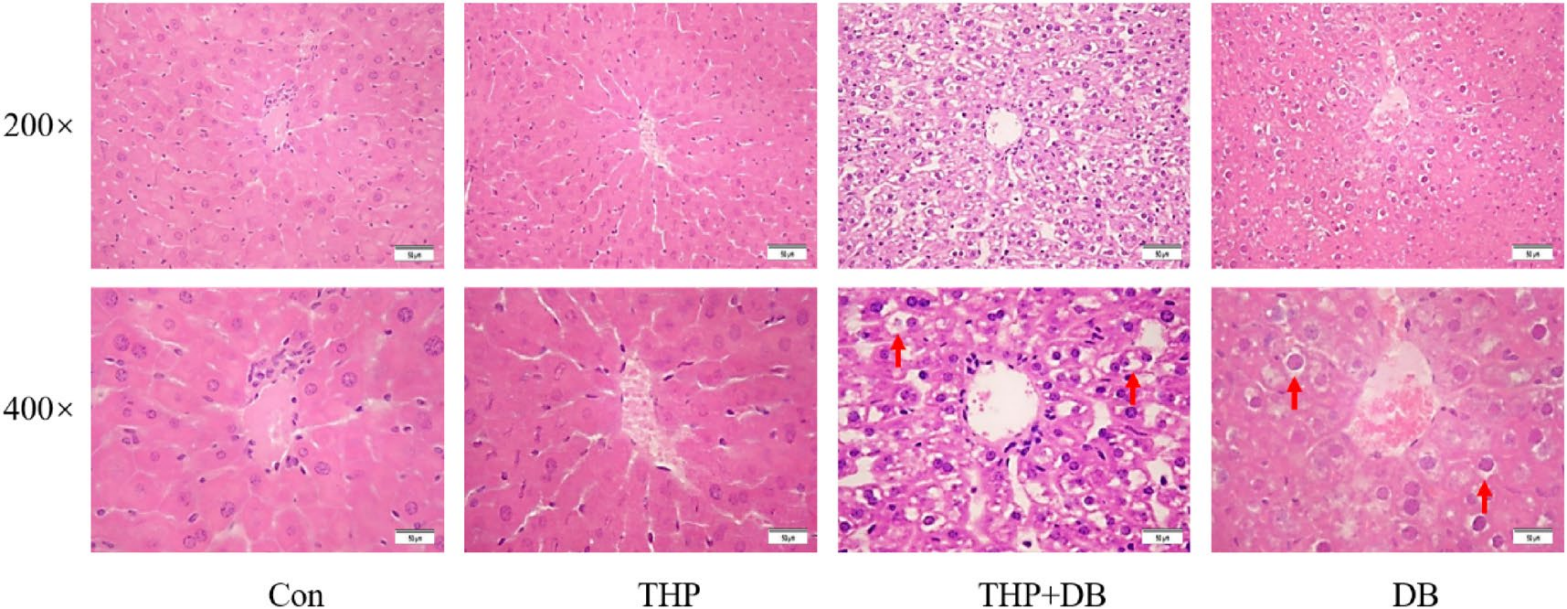
Morphology observation images (×200 and ×400) in mice livers (the morphological changes were indicated by an arrow). The livers of mice were stained with hematoxylin and eosin (HE). In THP and THP + DB groups, the normal structure of the hepatic lobules and the hepatic sinusoids had disappeared, the cytoplasm was loose and empty, balloon-like degeneration was visible. Con: Control group, THP: pirarubicin group, THP + DB: pirarubicin and DB extract group, DB: DB extract group.

In the control and THP groups, the hepatic lobules of the liver tissue were clear, the hepatocytes were arranged in an orderly manner, and the nuclei of the hepatocytes were clearly visible. In the THP and THP + DB groups, the normal structure of the hepatic lobules had disappeared, the hepatic sinusoids were absent, the hepatic cells surrounding the portal vein showed flaky necrosis, there were many infiltrating inflammatory cells, the hepatocytes were obviously edematous, the cytoplasm was loose and empty, and balloon-like degeneration was visible (Fig. 6). The damage of hepatocytes caused by DB extract was mainly embodied by the change of cytoplasm, which could not be reflected in the result of immunohistochemistry (Figs. 4, 5) due to the staining site of hematoxylin.

## Discussion

The use of herbal medicines as an important component of multicomponent therapy has increased steadily over the past decade. Eighty percent of people in Asian countries use herbs to promote health and treat common diseases, such as inflammation, pain, heart disease, cirrhosis, and central nervous system disease. However, with the increasing use of herbal medicines, the risk of HDIs has increased as well^13,14^. DB polysaccharides have antitumor properties in mice bearing U14 cervical carcinoma and strengthen the antitumor effect of cyclophosphamide^10^. However, DB extract can enhance the cardiotoxicity of THP, according to our study. Cardiotoxicity is the main adverse effect of THP. K S Sridhar et al. had reported that there was no cardiotoxicity with a cumulative dose of 300 mg/m^2^ THP^15^. The drug administration plan was based on the methods in literature and preliminary work of our group^16,17^. The dose of pirarubicin was not the clinical equivalent. In order to speed up the formation of cardiotoxicity induced by THP, mice were administrated with a doubled-dosage of THP single high dose which could develop cardiotoxicity after two days of administration. The muscle mass heart biopsies of the THP group showed extensive cardiovascular damage, muscle fiber breakage and necrosis in the THP + DB group according to the histopathology results. Combined DB extract and THP treatment showed a trend suggesting that cardiotoxicity could be strengthened by DB supplementation.

Next, we explored the mechanisms by which DB extract causes increased THP cardiotoxicity. Since the cardiotoxicity induced by THP is dose dependent, we hypothesize that the increased cardiotoxicity after combined use is due to the accumulation of THP in the body. The concentrations of THP in serum and heart were significantly elevated by the co-administration of DB extract and THP. THP efflux is mainly involved in P-gp, and the use of THP will lead to an increase in P-gp, which is also the reason for THP resistance. When a drug induces the function of efflux transporters, which accelerates the transport of the drug itself or a combination of other drugs out of the cell to reduce the toxic side effects of the drug on the cell, but it also reduces the efficacy of the drug. When a drug appears to inhibit efflux transporters, it will stay in cells and organs for a long time, enhancing the efficacy of the drug will also increase toxic side effects. P-gp and Mrp2 plays an important role in anthracycline efflux which can lead to drug accumulation if suppressed^18^. Because of the reduction of P-gp and Mrp2 in DB group, we hypothesized that THP accumulation was due to the inhibition of P-gp and Mrp2 expression by DB extract, which resulted in the accumulation of THP in vivo and increased cardiotoxicity.

There is high expression of P-gp in liver, kidney, brain and placenta^19^, which suggests that P-gp plays an important role in the excretion of exogenous substances and metabolites into the intestinal cavity, bile and urine and in preventing their accumulation in the body. Its high expression in the normal liver may influence the absorption and transport of drugs and affect their efficacy, which is one of the reasons for drug-drug interactions. Improper use of xanthate can cause multiple organ damage, such as liver damage and kidney damage, especially hepatotoxicity^20^. Niu et al. found that ethyl acetate extract could cause serious liver injury in ICR mice^21^. Similar to P-gp, Mrp2 is also one of the transporters involved in metabolism in the liver. Mrp2 can transport various substrates and is an important role in drug resistance in cancer^22–24^. The expression of Mrp2 by THP has not been reported in the literature.

We hypothesized that DB extract may lead to THP accumulation in the body via intrahepatic expression change of P-gp and Mrp2. According to the liver immunohistochemical results, THP significantly enhanced P-gp and Mrp2 -positive staining in the membrane and cytoplasm of mouse liver cells, and the P-gp and Mrp2 expression as detected by Western blot also increased. The expression of P-gp and Mrp2 in the DB group was lower than that in the control group, which indicated that DB could inhibit the intrahepatic P-gp and Mrp2 expression. In the THP + DB group, the positive staining of P-gp and Mrp2 decreased significantly, and the protein expression also decreased significantly. These results suggested that DB extract can significantly reduce the high expression of P-gp and Mrp2 induced by THP. As an efflux transporter of THP, the decreased expression of P-gp and Mrp2 lead to accumulation of THP, which increased the cardiotoxicity of THP. Thus, the downregulated intrahepatic expression of P-gp and Mrp2 led to a decrease in extracellular THP and then the accumulation of THP, which led to severer cardiotoxicity.

To explore the mechanism by which DB extract inhibits the intrahepatic protein expression of P-gp and Mrp2, we studied the morphological changes in liver cells in mice by immunohistochemistry. Many inflammatory cells infiltrated the hepatic tissue. It is suggested that the decrease in P-gp and Mrp2 expression by DB extract may be caused by the destruction of hepatocytes after liver injury. P-gp and Mrp2 may play an important role in HDIs between DB extract and THP. However, it is not clear whether DB extract is the substrate of P-gp and Mrp2 and further research of effect between DB extract and P-gp and Mrp2 is needed.

One limitation of this study is that we merely studied the effects of DB water extract on the cardiotoxicity of THP, instead of specific component of DB. However, DB water extract contains a number of components, including diterpene lactones, steroidal saponins, flavonoids, alkaloids, and micronutrients. Diterpene lactones are a large class of active and toxic components in DB extract that have anti-inflammatory, antibacterial and antitumor effects but also lead to hepatotoxicity. The component-induced HDI and the molecular mechanisms of DB extract on P-gp and Mrp2 activity and expression should be verified in the next phase.

**Figure 7:**
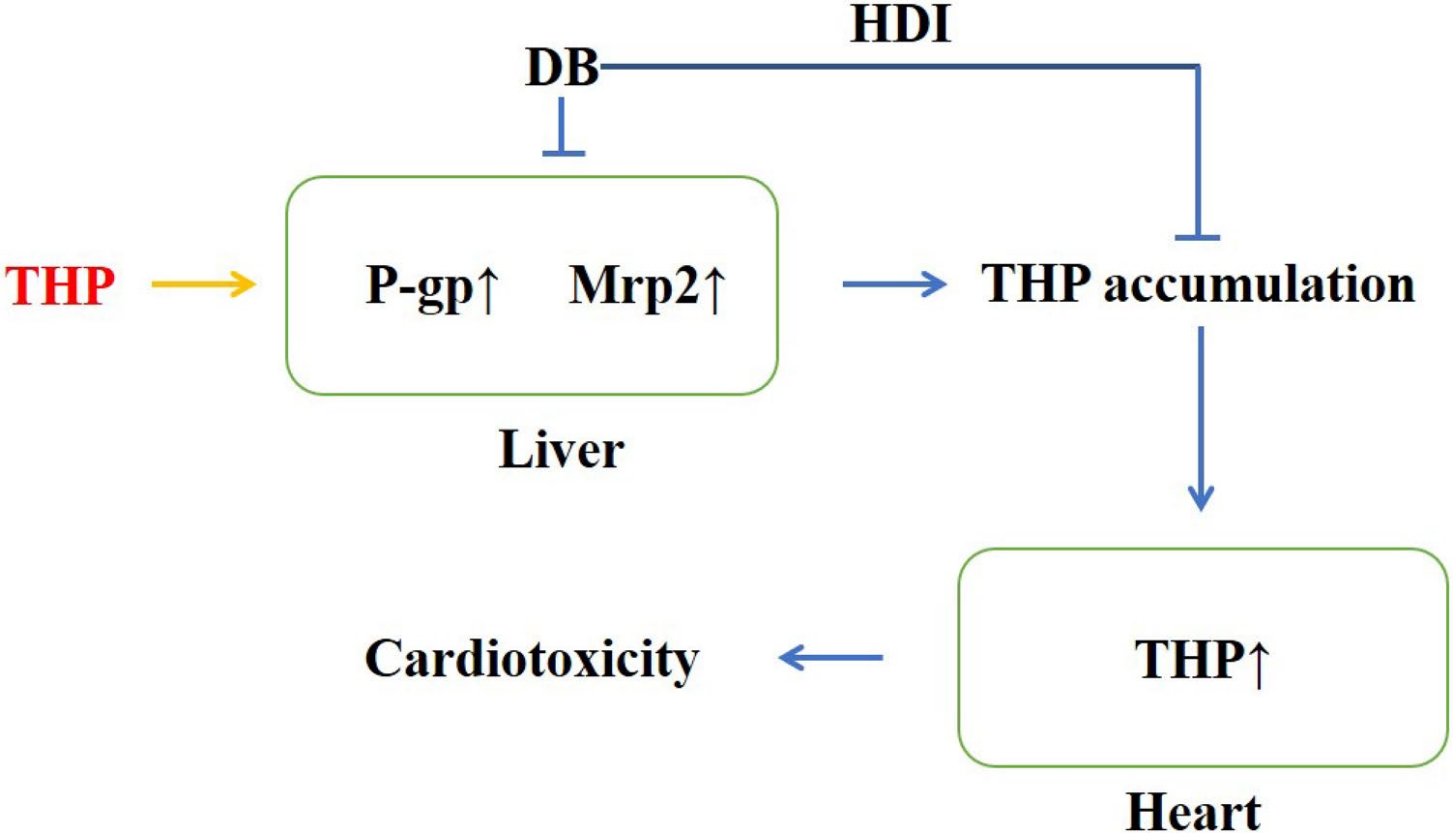
The mechanism of cardiotoxicity aggravated by co-administration of DB and THP.

In conclusion, the mechanism of HDI between DB extract and THP may be the hepatotoxicity of DB, leading to liver cell damage, downregulating the expression of P-gp and Mrp2, leading to a reduction in THP exocytosis, accumulation and aggravating cardiotoxicity (Fig. 7). In clinical it is necessary to avoid or monitor cardiotoxicity when DB extract and THP are co-administration.

## Materials and methods

### Animals

Male Kunming mice (20–25 g) were provided by the Laboratory Animal Center of Jilin University, and the environment temperature was 22 °C and the light and dark were alternated. Every five mice were in one cage and were given plenty of feed and water. There was a week for the mice to adjust to the environment before the experiments. All experiment procedures were approved by the Experimental Animal Ethics Committee of the First Hospital of Jilin University (No. 2019-238).

### Drugs

Crude dried DB were purchased from Anhui Province, China. THP for injection was purchased from HISUN PHARMACEUTICAL (Taizhou, China).

### Methods

All methods described in this work were performed in accordance with relevant guidelines and regulations of Laboratory Animal Center of the First Hospital of Jilin University.

### Preparation of DB water extract

The dried DB were crushed, and 100 g of DB pieces were soaked in water for 12 h. Then the pieces were extracted with water at 90 °C for 120 min. The extraction process was repeated for three times. All of the extracted solution was blended and filtered, and then inspissated at − 80 °C. Ultimately, each 300 mL of water contained the dissolved extract of 100 g of crude DB^12^. The quantification of DB water extract and marker ingredient Diosbulbin B was analyzed by high-performance liquid chromatography (HPLC). The concentration of Diosbulbin B in DB solution sample was 1.26 mg/mL^12^.

### Drug administration and tissue collection in mice

Male Kunming mice (22–25 g) were randomly divided into four groups (control group, THP group, THP + DB group and DB group) with 10 mice in each group. The THP + DB and DB groups were given aqueous DB solution (0.1 mL/10 g) orally once daily for 14 consecutive days. Control (Con) and THP groups were given vehicle (water). On Day 12, the mice in THP and THP + DB groups were injected with THP (20 mg/kg) intraperitoneally. Control and DB groups were intraperitoneally injected with 0.9% NaCl on Day 12^11^. The mice that died during the experiment period were excluded and there was no mouse excluded in the experiment. On the 14th day, blood was collected from mouse eye sockets. The heart and liver tissues were collected and partially fixed in 4% paraformaldehyde for morphology observation and immunohistochemistry. The rest of tissues were stored at − 80 °C (Fig. 2A).

### Morphology observation

The livers and hearts of mice were washed and placed in 4% formaldehyde and immersed in paraffin, then cut into 5-micron-thick slides to stain with hematoxylin and eosin (HE) and examined with an optical microscope (Olympus, Japan)^25^.

### High performance liquid chromatography-mass spectrometer (HPLC–MS)

The concentrations of THP in serum and heart were detected by HPLC–MS analysis using a 1200 HPLC (Agilent, USA) and an API4000Q MS (AB Sciex, USA) equipped with an electrospray ionization (ESI) source. Chromatographic column was SB C18 columns. The composition of mobile phase was acetonitrile and water with 0.1% formic acid and the flow rate was 0.25 mL/min. The column temperature was 30 °C (Table 1)^12^. The internal standard was daunorubicin.

**Table 1:** Mobile phase condition.

Time (min)	Acetonitrile (%, v/v)	Water with 0.1% formic acid (%, v/v)
0.0	20	80
2.0	20	80
6.0	90	10
8.0	90	10
8.1	20	80
12.0	20	80

The MS was accomplished in positive ionizational multiple reaction monitoring (MRM) mode. The optimum values of MS were as follows: ionspray voltage, 5.5 kV; desolvation gas flow pressure, 0.75 MPa; nebulizer gas flow pressure, 0.625 MPa; source temperature, 550.0 °C.

### Immunohistochemistry

Livers of mice were embedded in paraffin and cut into sections. The sections were incubated with rabbit anti-P-gp, rabbit anti-Mrp2 (Santa Cruz Biotechnology, Texas, USA) at 4 °C overnight, then incubated with horseradish peroxidase-labelled secondary antibody. After coloration the sections were observed with a BX51 optical microscope (Olympus, Japan).

### Western blot

Mouse livers were homogenized in RIPA buffer (1% Triton X-100, 1% deoxycholate, 0.1% SDS) and centrifuged at 12,000* g* for 5 min at 4 °C. The supernatant was collected, and total protein was quantified using the Bradford method. Cell lysates contained 50 μg of protein per lane were separated by 10% SDS-PAGE and electrotransferred onto PVDF membranes. The membranes were blocked with TBST buffer for 1 h at room temperature. The membranes were then incubated overnight at 4 °C with primary antibodies for 2 h. Then the membranes were washed and incubated with secondary antibodies for 1 h at room temperature. Immunoreactive proteins were visualized using an electro-chemi-luminescence (ECL) system, and the band density was analyzed using ImageJ software (Bethesda, MD).

### Statistical analysis

All data were described as the mean values ± standard deviation (SD) and the analysis was done with SPSS 18.0 software (https://dl.pconline.com.cn/download/1118212-1.html and the version number is 18.0.0). Differences between groups of HPLC–MS experiments were assessed by Student’s *t*-test after the data were confirmed to meet the assumptions for those tests. And statistics of data in the Western blot were performed with Two-way ANOVA.

## Supplementary Information

Supplementary Information.
