# Supplementary material for: Extract from Dioscorea bulbifera L. rhizomes aggravate pirarubicin-induced cardiotoxicity by inhibiting the expression of P-glycoprotein and multidrug resistance-associated protein 2 in the mouse liver
Source: Sci Rep. 2021 Oct 5;11:19720. doi: 10.1038/s41598-021-99264-2 (PMC8492811; doi:10.1038/s41598-021-99264-2)
Supplement: Supplementary file 1 — Supplementary Information. [file 41598_2021_99264_MOESM1_ESM.pdf]

Extract from *Dioscorea bulbifera* L. Rhizomes Aggravate Pirarubicin-Induced Cardiotoxicity by Inhibiting the Expression of P-Glycoprotein and Multidrug Resistance-Associated Protein 2 in the Mouse Liver

Li-rui Sun[1], Qiu-shi Guo[1], Wei Zhou[1] & Min Li[2,*]

1 Department of Pharmacy, the First Hospital of Jilin University, Changchun, Jilin, China.

2 Pharmacological Experiment Center, School of Pharmaceutical Sciences, Jilin University, Changchun, Jilin, China. *email: susanna@jlu.edu.cn

Supplement

Samples	Concentration (μg/mL)	Recovery (%)	Intra-day precisions (RSD)	Inter-day precisions (RSD)
Serum	0.8	96.3±0.8	3.3	6.4
	5.0	99.2±1.1	3.1	5.2
	15.0	97.8±0.6	2.1	3.7
heart	0.8	96.2±2.2	4.0	7.2
	5.0	97.4±1.7	3.4	6.6
	15.0	98.3±2.3	3.5	4.9

Supplementary **Table 1**. The results of precision and accuracy of THP in mice serum and heart (n=6)

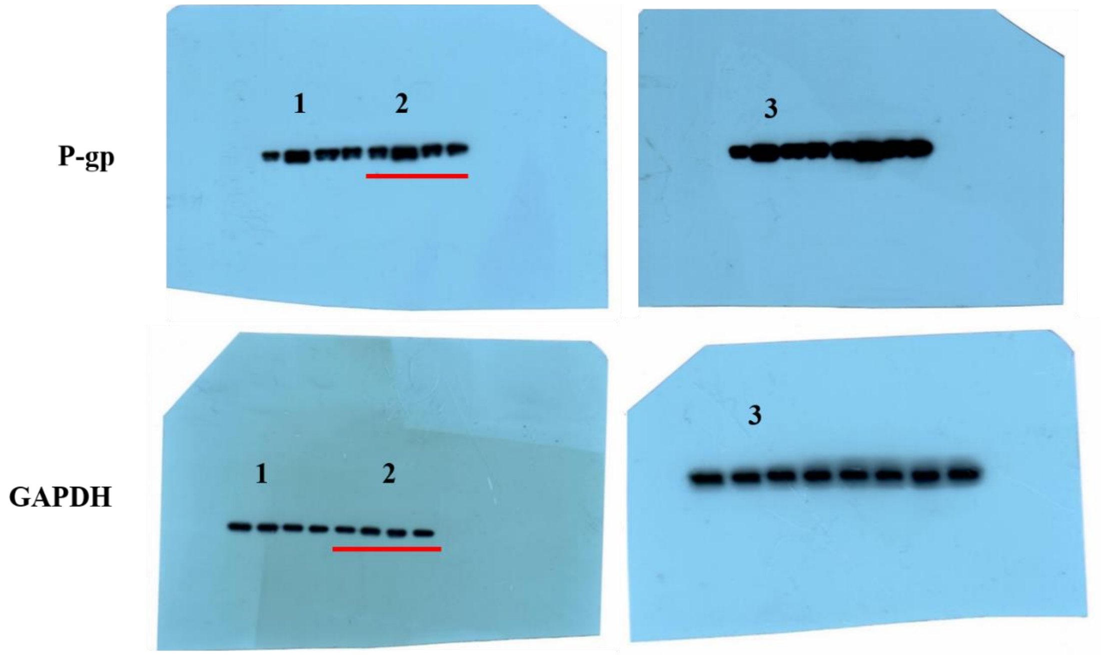

Supplementary Figure 1. The original blot image of P-gp and GAPDH. Bands in one group of P-gp were not separated very well and we used the remaining three groups of results for statistical analysis. The bands used in Figure 4B were marked with a red line.

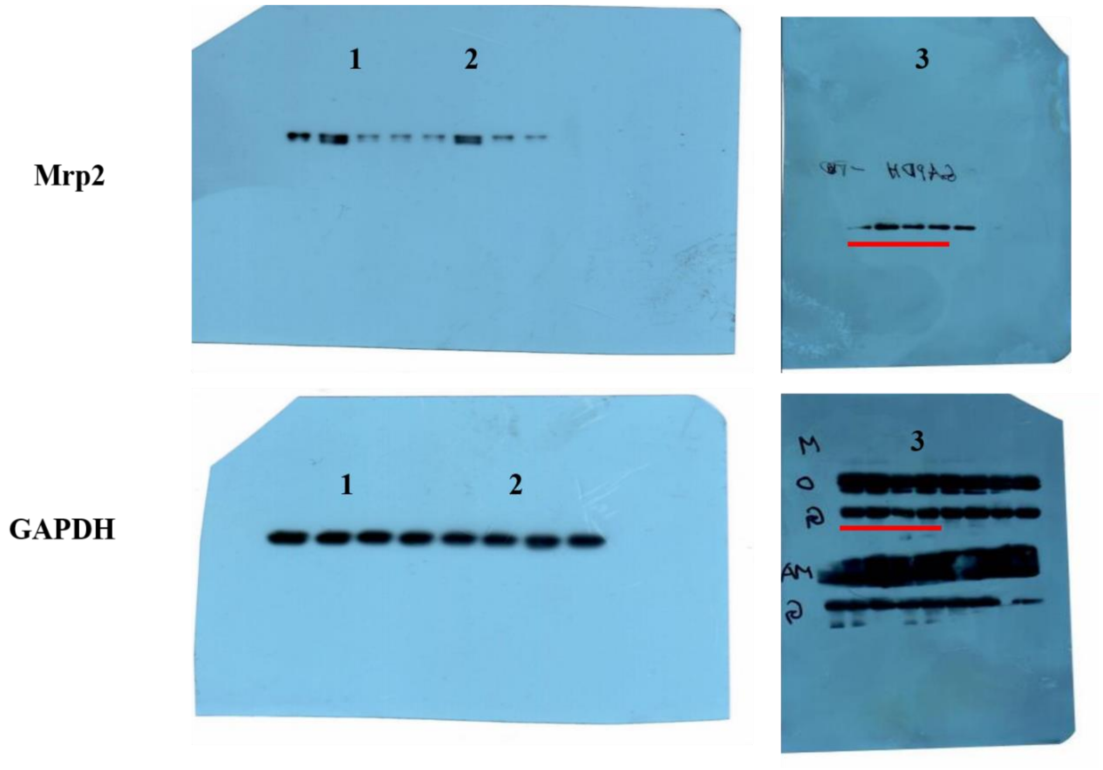

Supplementary Figure 2. The original blot image of Mrp2 and GAPDH. Bands in one group of Mrp2 were not developed very well and we used the remaining three groups of results for statistical analysis. The bands used in Figure 5B were marked with a red line.
